# Outcomes of Femoral Artery Pseudoaneurysm in Intravenous Drug Abusers Managed at a Tertiary Care Center

**DOI:** 10.7759/cureus.13350

**Published:** 2021-02-15

**Authors:** Najam U DDin, Fahad Tariq Berlas, Khalil Ur Rehman, Ghulam Ali, Farhina Salahuddin, Asma Mumtaz

**Affiliations:** 1 Department of Vascular Surgery, Shaheed Mohtarma Benazir Bhutto Institute of Trauma, Karachi, PAK

**Keywords:** femoral artery pseudoaneurysm, ligation, intravenous drug user, excision, vascular complication, vascular surgery, intravenous drug abusers (ivda), pseudoaneurysm

## Abstract

Objectives

The aim of this study was to assess the effects of ligation and excision of femoral artery pseudoaneurysm without revascularization in intravenous drug abusers presenting in the tertiary care center.

Methods

This was a retrospective study conducted at Shaheed Mohtarma Benazir Bhutto Institute, Karachi, Pakistan, and included 119 patients admitted for vascular injuries of the groin between June 2016 and June 2020. Data collected from the hospital's medical records included all intravenous drug addicts presented with mass near or at groin area only, while other pseudoaneurysm locations secondary to vascular trauma, arteriovenous fistula, and hemodialysis were excluded. SPSS Version 20.0 (IBM Corp., Armonk, NY, USA) was used for data analysis.

Results

This study included 119 patients, all of whom presented and admitted to the Accident and Emergency Department, with a mean age of about 32 years ± 11.34 years and a mean duration of addiction of 2.47 years ± 1.37 years. Males constituted 83.2% of the patients, while females constituted 16.8%. The left femoral artery was affected more commonly than the right femoral artery, with an average of 75.6% and 24.4%, respectively. The most common presentation was bleeding from ruptured pseudoaneurysm (76.5%) and oozing with pulsatile mass (17.6%), while infected pulsatile swelling and misdiagnosis were uncommon. After surgical intervention, limb salvage was 95.8%, whereas mortality and amputation rate were 2.5 % and 1.7%, respectively.

Conclusion

The optimal management of femoral artery pseudoaneurysm in intravenous drug addicts is ligation and excision of the pseudoaneurysm without revascularization.

## Introduction

The world is facing a surge in a contagious addiction to illicit drug abuse among the nations' youth. Though no current national data are available for an accurate estimate of drug addiction among Pakistanis, around 7.8 million people are drug addicts than a global estimated 35 million [1]. This illicit drug addiction poses a significant impact on a nation's economic and public health care. Overdosage, and local and systemic complications are frequent but late presented, endangering an individual's life, and sharing needles lead to acquiring hepatitis B/C and human immunodeficiency virus (HIV) infections [2].

After compromising their superficial veins, intravenous drug users (IVDUs) find easy access to their groin for the femoral vein, where they accidentally puncture the femoral artery leading to a pseudoaneurysm formation [3]. Repeated injury to the vessel wall leads to the formation of hematoma in surrounding tissue, resulting in the development of a pseudoaneurysm (false aneurysm), which is distinguished from true aneurysm as it lacks all three typical elements of the arterial wall [4]. The typical femoral artery pseudoaneurysm presentation includes a pulsatile mass, infection, and oozing of blood, with or without peripheral vascular compromise, and most lethal is massive bleeding [5].

Controversies in the management of femoral artery pseudoaneurysm are still prevalent around the globe. Nevertheless, primary repair following ligation and excision of pseudoaneurysm has been reported in the literature with acceptable results [6,7]. However, we preferably chose simple ligation of pseudoaneurysm, excision, and debridement of the surrounding tissue.

A recent study in Pakistan revealed intermittent claudication and amputation rate following the above treatment as 8% [8]. This study's primary purpose was to examine the outcome of ligation of pseudoaneurysm, excision, and debridement of the surrounding tissue without immediate revascularization in our institute.

## Materials and methods

This retrospective analysis was conducted at the Department of Vascular Surgery at Shaheed Mohtarma Benazir Bhutto Institute, Karachi, Pakistan. After institutional approval, 119 patients presented to the Emergency Department with femoral artery pseudoaneurysm and underwent surgical treatment for vascular injuries due to intravenous drug abuse.

The data of interest were obtained from hospital medical records, including patient's demographics (age, sex), site of injury, duration of addiction, laboratory investigations (hepatitis B, hepatitis C virus [HCV], and HIV screening), and post-operative complications/outcomes (limb salvage, claudication, threatened limb ischemia, amputation, and mortality). The pseudoaneurysm diagnosis was confirmed with a detailed history, examination, and color Doppler ultrasound routinely by one team member. Patients with pseudoaneurysm secondary to trauma, hemodialysis, arteriovenous fistula, and sites besides groin, and those who underwent primary revascularization were excluded.

Procedure

**Figure 1:**
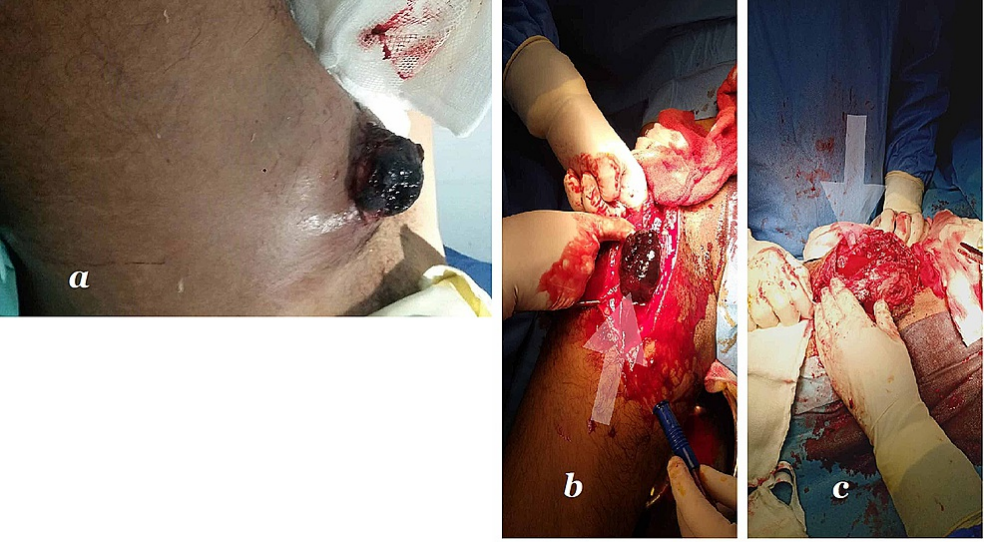
(a) Presentation at the Emergency Department. (b, c) Intra-operative exposure of femoral artery pseudoaneurysm

The surgical technique included a proximal ligation of the distal part of the external iliac vessels followed by femoral arteries through uninfected fields. The mass was excised, and debridement of the surrounding local tissue was performed (as it mostly has local infected surroundings). The wound was irrigated with saline and left open for secondary healing with the use of negative pressure (Figure 1).

The total hospital stay time was of about two weeks till the stitches were removed. Outcomes of surgical intervention were determined during this period.

Statistical analysis

The data analysis was performed using SPSS Version 20.0 (IBM Corp., Armonk, NY, USA), with a p-value of <0.05 deemed statistically significant. Mean and standard deviations (SDs) were calculated for quantitative data, while frequency and percentages were calculated for qualitative data. The data were stratified for age, sex, co-morbidities, duration of addiction, mode of presentation, side of the limb, and outcome variables.

## Results

A total of 119 patients presented to the Emergency Department with femoral artery pseudoaneurysm secondary to intravenous drug abuse and underwent surgical management. The mean age was 32 years (SD: 11.34 years). Out of 119 patients, 99 (83.2%) were males and 20 (16.8%) were females. All patients admitted to the Emergency Department, with the left femoral artery involvement about three times more common than its counterpart. The mean duration of addiction was 2.47 years (SD = 1.37 years), as shown in Table 1.

**Table 1:** Descriptive statistical analysis OPD, outpatient department

Variables (n=119 )	Mean ± SD/frequency
Age (years) (18-70 years)	31.94 ± 11.34
Gender	
Male	99 (83.2%)
Female	20 (16.8%)
Mode of admission	
Emergency	119 (100%)
OPD	0
Side of injury	
Left	90 (75.6%)
Right	29 (24.4%)
Duration of addiction (years)	2.47 ± 1.37

**Figure 2:**
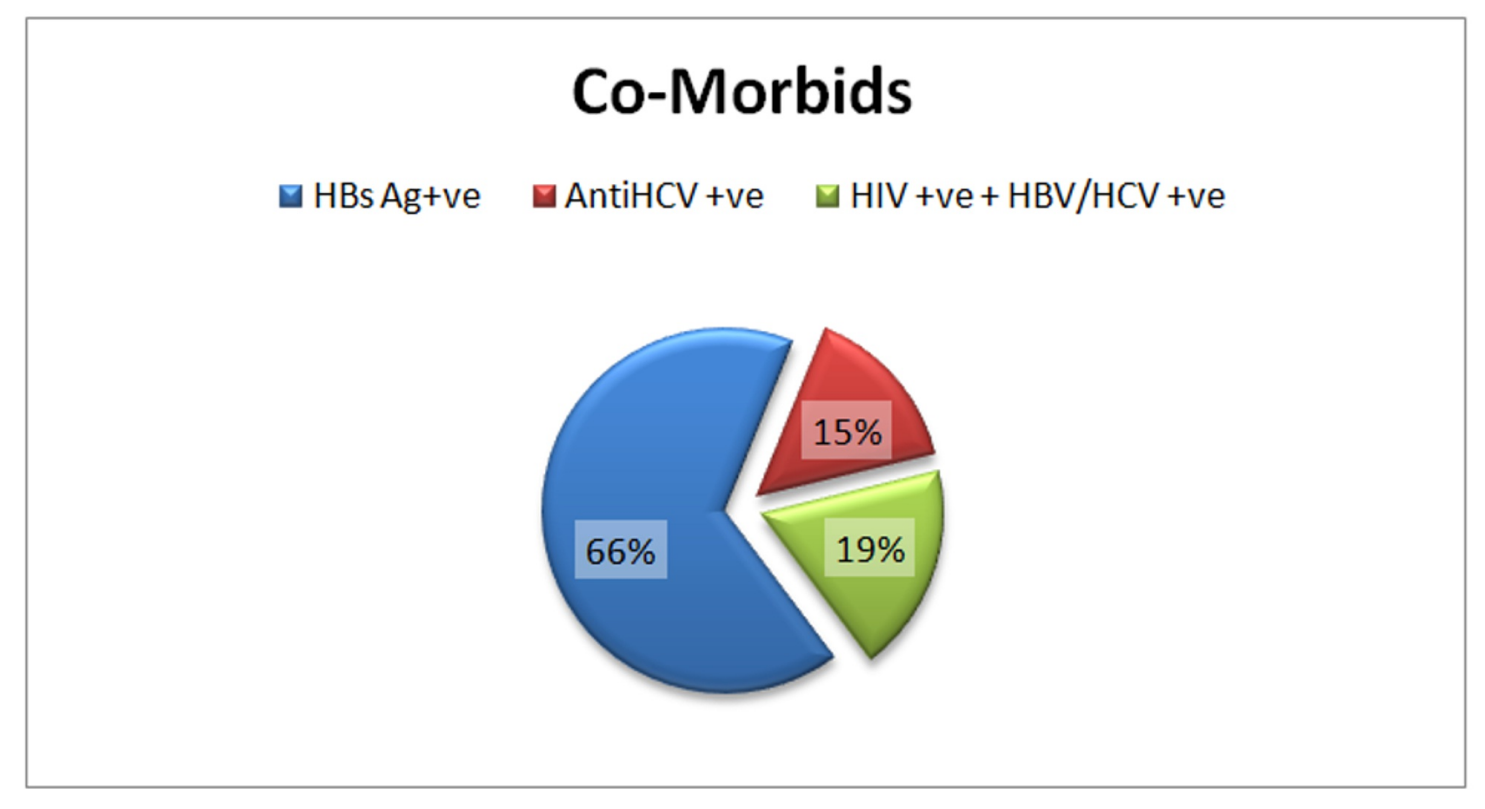
Co-morbids HBs Ag, hepatitis B surface antigen; HCV, hepatitis C virus; HIV, human immunodeficiency virus; HBV, hepatitis B virus

All patients had chronic infections, with the far more prevalent being hepatitis B virus (66%) followed by a combined infection of hepatitis B/C + HIV (19%) and HCV only (15%) (Figure 2).

**Figure 3:**
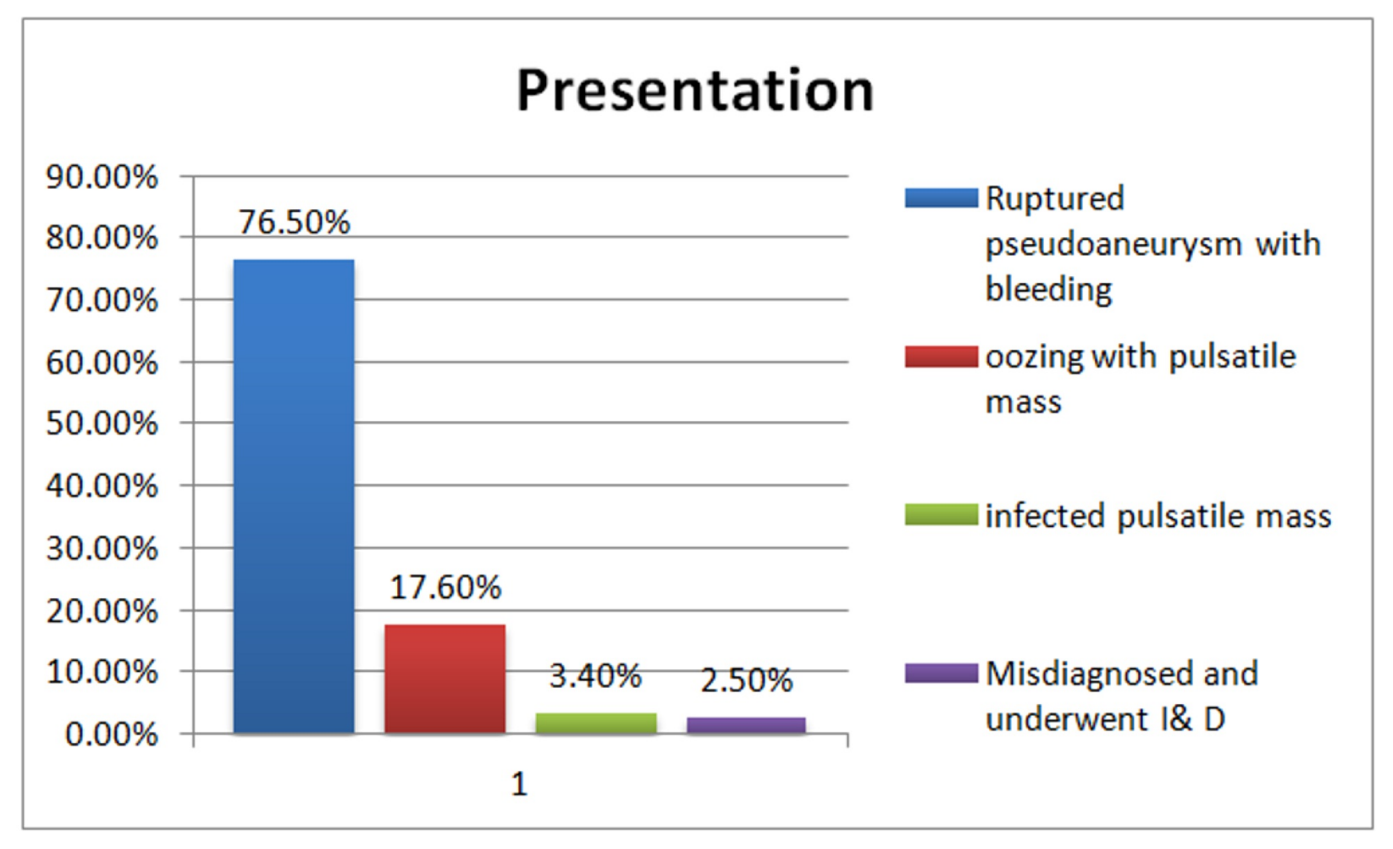
Presentation

Ruptured pseudoaneurysm (76.5%) and oozing with pulsatile mass (17.6%) were the most common presentation in the Emergency Department, with less frequent infected pulsatile swelling misdiagnosis being a rare presentation, as shown in Figure 3.

All patients underwent ligation, excision, and debridement of the surrounding tissue (if infected) without primary revascularization. Following this procedure, only two (1.7%) patients developed limb ischemia, for which amputation was performed. Overall, the mortality rate was 2.5%, while a limb salvage rate of 95.8% was recorded (Table 2).

**Table 2:** Outcome

Variables (n=119 )	Mean ± SD/Frequency
Outcome	
Limb salvage	103 (95.8%)
Threatened limb ischemia	0 (0%)
Amputation	2 (1.7)
Mortality	3 (2.5)

## Discussion

Pseudoaneurysm (false aneurysm) results from prolonged, unintended, non-sterilized, peri-arterial, repeated injuries to the blood vessels for narcotics administration. Femoral vessels in the femoral triangle are convenient routes for drug administration due to their superficial position. Vascular injuries in drug abusers are common in this area followed by brachial and other vessels [4,5]. The management of pseudoaneurysm is quite a dilemma for vascular surgeons around the globe. Late presentation, compromised vessels (for autogenous graft), infection, mental addiction disorders, loss of follow-up, and lack of compliance with medication advice are the main reasons behind unfavorable outcomes. These patients also present later with the same problem but at different locations.

In our hospital ( Shaheed Mohtarma Benazir Bhutto Trauma Center), which is the only public sector hospital and primary referral hospital providing vascular surgery services to the major bulk of the country population, 119 patients who had groin vascular complications in intravenous drug use have been treated so far. The most common presentation is ruptured pseudoaneurysm with bleeding (76%), which is consistent with that reported in the literature (40-70%) [3,8,9]. Most of the patients in this study were young (20-40 years), with males being dominant compared to other reviews [3,10], with a male-to-female ratio of 4:1. Female drug addiction is not common, and its prevalence has been increased recently in our society.

IVDUs are infected frequently with blood-borne viruses. Serological results are often unavailable at the surgery, and therefore strict precautions should be implemented in these particular patients. In these neglected patients, infections such as hepatitis C, hepatitis B, and HIV are rising [2,3,11]. In this study, infection with hepatitis B is more prevalent (66.4%) followed by a combined infection of HIV and either hepatitis B or C viruses (18.5%) and hepatitis C alone (15.1%).

Pseudoaneurysm ligation, resection, and thorough debridement of the surrounding tissue had been successfully applied as most of the groin pseudoaneurysms were infected. The only drawback of this treatment is severe claudication and threatened limb ischemia requiring amputation later on. Controversies are prevalent in the decision-making of the best strategy for surgical management of this complicated vascular problem. Most surgeons perform revascularization in fear of limb loss. In our study, the ligation of pseudoaneurysm followed by excision without revascularization was performed. An investigation revealed a higher amputation rate, i.e., >35, following arterial ligation, which is suggestive of a higher risk of amputation than previously published. However, in this study, ligation was higher than in our study, explaining this higher rate [12]. Conversely, in this study, three patients died due to sepsis and thromboembolic conditions, and two patients required amputation in index admission due to delayed presentation.

The most extensive single-center research on pseudoaneurysm management in IVDU has reported no limb loss but forefoot amputation only [13]. The only possible explanation for the absence of limb ischemia in these patients is the development of collateral circulation due to progressive enlargement of a pseudoaneurysm [9]. In contrast to the above, primary revascularization with autologous or prosthetic grafts had documented reinfection, graft disruption, limb loss, and even mortality [6,14-16].

A similar study has been conducted on brachial artery pseudoaneurysm in our department, which shows promising results of the ligation and excision of the pseudoaneurysm without revascularization among IVDUs [3]. This study also reveals around 96% of limb salvage by this procedure, which ensures generalizing this treatment strategy in emergency vascular complications in IVDUs. The absence of proper follow-up to evaluate this procedure's outcome is the only limitation of this study. Patients do not follow the guided help and may find ways back to addiction.

## Conclusions

This study provides strong evidence to support simple ligation and excision of femoral artery pseudoaneurysm without revascularization, an effective treatment option in the emergency. In comparison, others dangle in the controversies of revascularization. Nevertheless, studies with long-term follow-up are required to standardize this treatment in our institute.
